# Improvement of the occupational risk management process in the work safety system of the enterprise

**DOI:** 10.3389/fpubh.2023.1330430

**Published:** 2024-01-15

**Authors:** Oleg Bazaluk, Vitalii Tsopa, Małgorzata Okrasa, Artem Pavlychenko, Serhii Cheberiachko, Olena Yavorska, Oleg Deryugin, Vasyl Lozynskyi

**Affiliations:** ^1^Belt and Road Initiative Center for Chinese-European Studies (BRICCES), Guangdong University of Petrochemical Technology, Maoming, China; ^2^Department of Management and Economics, International Institute of Management, Kyiv, Ukraine; ^3^Department of Personal Protective Equipment, Central Institute of Labour Protection – National Research Institute, Łódź, Poland; ^4^Department of Ecology and Technologies of Environmental Protection, Dnipro University of Technology, Dnipro, Ukraine; ^5^Department of Labour Protection and Civil Safety, Dnipro University of Technology, Dnipro, Ukraine; ^6^Department of Transportation Management, Dnipro University of Technology, Dnipro, Ukraine; ^7^Department of Mining Engineering and Education, Dnipro University of Technology, Dnipro, Ukraine

**Keywords:** occupational safety, occupational risk, assessment of occupational risk, hazardous factor, “BOW-TIE” method

## Abstract

**Purpose:**

The research purpose is to improve the management of occupational risks associated with hazards as well as the organization’s capabilities to identify hazardous factors (HFs) using the “BOW-TIE” method in accordance with the provisions of the ISO 45001:2019 standard.

**Methods:**

To improve occupational risk management, the “BOW-TIE” method has been introduced into occupational health and safety management systems. This approach facilitates a comprehensive description and analysis of potential risk development from identifying hazardous factors to studying the consequences. It visually integrates fault and event trees to provide a holistic view of risk dynamics.

**Results:**

The improvement of the occupational hazard risk management process considers both internal and external factors affecting the organization, thereby increasing the probability and severity of potential hazardous events. The revised approach categorizes risk levels as acceptable, unacceptable, or verifiable. In addition, occupational risk management requires an in-depth analysis of the organization’s external and internal environment to identify hazards that affect the probability and severity of potential hazardous events.

**Conclusion:**

This research proposes an innovative approach to occupational risk management by determining the magnitude of occupational risk as the cumulative result of assessing risks associated with all external and internal factors influencing the probability of hazardous event occurring. The introduction of the “BOW-TIE” method, combined with a comprehensive analysis of the organizational environments, facilitates a more effective and nuanced approach to occupational risk management.

## 1 Introduction

For the development of a country and society as a whole on the way to joining the European Union, it is necessary to ensure the implementation of production processes with the least expenditure of effort and resources, together with high occupational safety and health of employees (1–4).

The model of any business for managing losses, including losses of life and health of workers, can be presented in the form of a list of external and internal hazardous factors (hereinafter – HF): social, environmental, economic, industrial, innovative, scientific, and technical and other, which are interconnected and contribute to the improvement or deterioration of business process management systems at enterprises under certain conditions (5).

Under certain conditions, the cumulative impact of all hazardous factors will contribute to the deterioration of the effectiveness of business process management systems at enterprises (6, 7).

Therefore, their analysis, identification of the most influential ORs, or their combinations is the basis for making any decisions based on occupational risk (OR) assessment in occupational safety and health management systems to substantiate protective and precautionary measures to reduce injuries when performing production tasks (8–11). It is this approach that makes it possible to ensure the organization’s resilience to the impact of destructive factors and reduce the impact of negative consequences and financial losses (12).

In accordance with the requirements of the ISO 45001:2018 standard (13), each enterprise must determine all external and internal HFs of organization environment that can increase the probability of the occurrence of hazards occurring in occupational safety and health management systems that will not achieve the intended result, reducing injuries and occupational diseases in the organization. In order to identify all the hazardous external and internal factors, the SWOT analysis is the basic one (14). If additional consideration of external groups of HFs (political, economic, social, etc.) is necessary, PEST analysis is added. To identify neutral HFs, which can be either negative or positive, requiring additional analysis, the SNW analysis method is used (Table 1).

**Table 1:** Methods of HF analysis of the enterprise environment.

Method	Description of the method
SWOT analysis (15)	SWOT analysis is a method of strategic planning, which consists in identifying the HF of the internal and external environment of the enterprise. The task of the SWOT analysis is to give a structured description of the situation in relation to which a decision must be made. The conclusions drawn on its basis are descriptive in nature without recommendations and prioritization.
PEST- analysis (16, 17)	The method is designed to identify political *(political)*, economic *(economic)*, social *(social)*, and technological *(technological)* aspects of the external environment that affect the company’s business. Varieties: PESTLE analysis, SLEPT-analysis, STEEPLE- analysis, STEEP, PESTEL, PESTELI, LONGPEST.
SNW analysis (18)	SNW analysis (strength, neutral, and weakness) is a method of analysing the company’s strengths, neutrals, and weaknesses. The purpose of this process can be considered to be the identification of advantages, and then - the elimination of shortcomings among them and their strengthening.

At the same time, there is a challenge in identifying the HF impact on the level of occupational risks, the value of which forms awareness of the impact of uncertainty on the objectives set in the relevant management systems (19).

It is a common understanding that the risk assessment process focuses only on the analysis of the potential threat and its consequences. In this case, to determine the cause-and-effect relationship between the hazard and hazardous event, focus is made exclusively on one factor (20, 21).

With this approach to risk assessment, a complete understanding of the structure of the environment in which the enterprise operates is often lost. This does not allow to reflect all possible influences on the OR of hazards (probability and degree of severity of a hazardous event: accident, failure, etc.) at employees’ workplaces (22, 23). Hence, the need arises to conduct appropriate scientific research to develop new or improve known OR management approaches that would allow taking into account the cumulative impact of any internal and external HFs of any enterprise organization.

There are 32 different methods for analyzing the OR management process in an enterprise environment, each having its own strengths and weaknesses (24). However, there is always a question about the effectiveness of known methods for solving the tasks in a particular enterprise. The authors of the publication (25) concluded that in order to choose the best method, first of all, it is necessary to understand what results the company wants to achieve. Because none of the known methods for analyzing the influential HF of the general environment of the enterprise, used in isolation, gives a complete picture of the structure that functions in enterprises, errors occur in management decisions aimed at improving security. However, the study considers the solution of a specific case of environmental impact assessment, which does not allow the developed approach to be applied to other conditions. At the same time, the authors of the scientific study (26) recommend using the results combining several methods to obtain the most complete information about hazards during production operations. However, unfortunately, no recommendations were made how to combine or select them. As the authors of the study (27) noted, in order to promote the progressive, inclusive, and sustainable economic growth of the enterprise, it is necessary that the enterprise management system, which covers the entire set of interrelated elements, is aimed precisely at eliminating all major threats and challenges it faces. At the same time, the biggest problem arises when making an effective decision due to the fact that it is difficult to determine the probability of occurrence of hazardous events from various threats when employees perform production activities (28).

Sometimes this requires quite serious scientific research. However, quite a few enterprises can afford to finance such projects. Moreover, the answer will be received after a certain period of time that is usually lacking. The specified task requires a clear understanding of the action of all possible hazards and HF, which can increase the probability of the occurrence of a hazardous event (29). We should note that the most popular for modeling hazardous processes, including accidents, were diagrams of cause-and-effect relationships, which received the names “failure tree” and “event tree” (30, 31). By getting all the information about the negative external or internal HF of enterprise, it is possible to understand the hazards in this or that scenario; it is possible to estimate the OR. If the OR is not acceptable, it should be reduced; that is, measures should be implemented to reduce the probability of occurrence of a hazardous event and/or the degree of severity of the consequences of a hazardous event. However, in most of the analyzed publications (32–35) regarding the assessment of OR, there is a misunderstanding between hazard and external and internal HF, which have a significant impact on the probability of the occurrence of a hazardous event and the severity of the consequences for the life and health of employees being a condition for OR changing (increasing or decreasing) (36). Therefore, there is a misunderstanding regarding measures to reduce the probability of the occurrence of a hazardous event and its severity since it is sometimes not possible to eliminate the hazard itself.

The purpose of the study is the improvement of the enterprise’s OR risk management process taking into account the requirements of the standard ISO 45001:2019.

## 2 Materials and methods

The most common approach to OR management, which is the basis for developing an action plan to achieve the desired result in the operation of the OHSMS system of any organization, is the “BOW-TIE” method. This method provides, on the basis of a cause-and-effect relationship between hazard and a hazardous event, and it allows to describe and analyze occupational risks during any production operations (37, 38).

**Figure 1:**
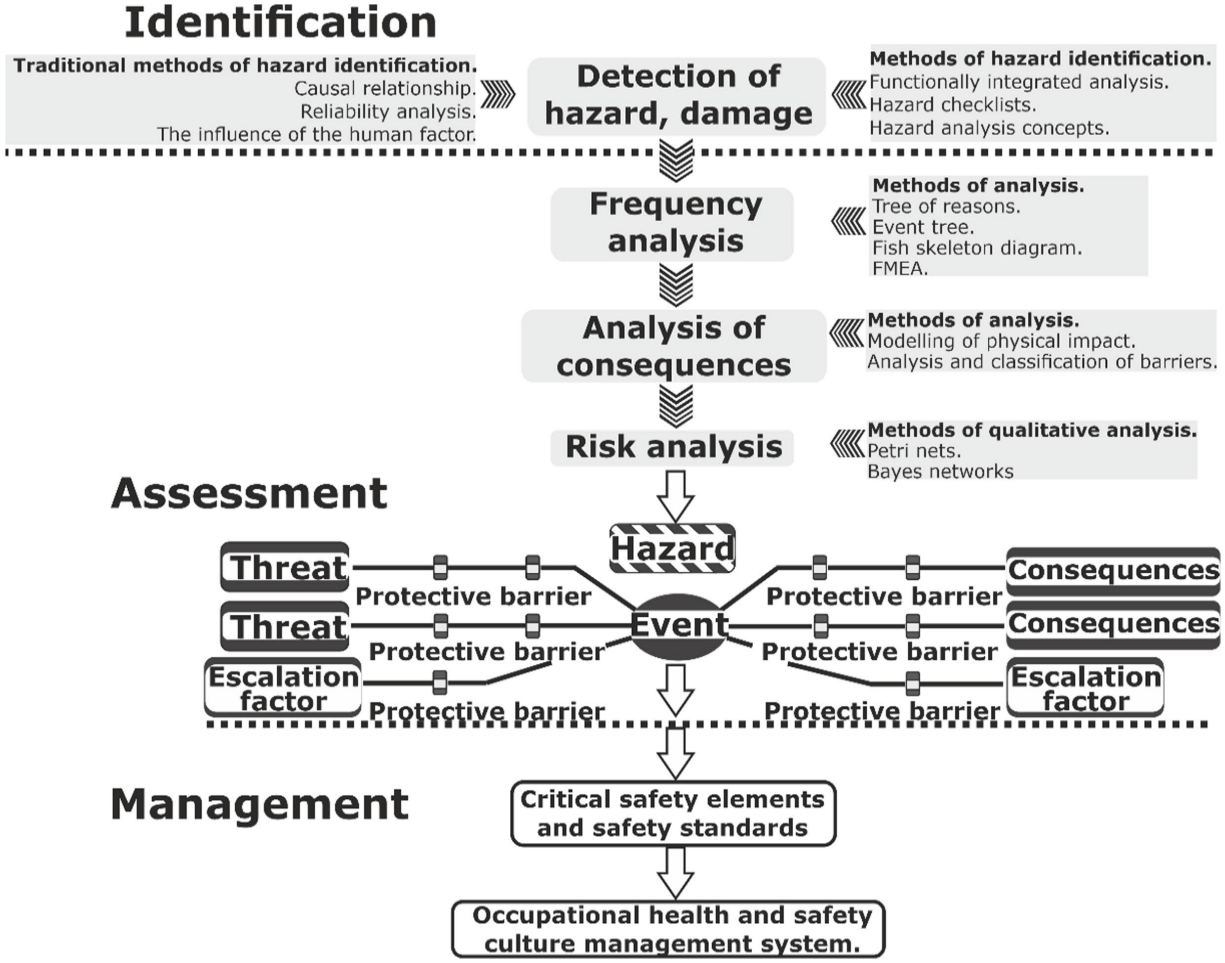
BOW-TIE classic method (37, 38).

It can also be used to determine the effectiveness of proposed “barriers” (precautionary or protective measures) to reduce the probability of a hazardous situation on the one hand, and on the other hand, the severity of the consequences of an incident (Figure 1).

In general, the presented method provides for the calculation of the amount of occupational risk:

- Identification of hazards for the development of their register in the organization based on the study of sources of risk.
- Studying the mechanisms of the development of a hazardous event based on a cause-and-effect relationship.
- Determination of the probability of the occurrence of a hazardous event - due to the processing of statistical data on all incidents that occurred anywhere.
- Determining the severity of losses, calculating the number of days of incapacity for work or coefficients of the severity of injuries, economic losses.
- Calculation of occupational risk—by any suitable method, which is defined in IEC 31010:2019 risk management—risk assessment techniques.

Unfortunately, the well-known interpretation of the described method does not take into account the influence of internal and external hazardous factors on the probability of a hazardous event, information about which is collected in the organization by one of the above approaches (Table 1). As a result, there is a lack of clear understanding of what affects the probability of a hazardous event and its degree of severity. At the same time, the ISO 45001:2018 standard mentions the need to determine external and internal factors that affect the final result of the occupational safety and health management system, i.e., reducing the loss of health and life of workers. The standard requires the organization to use these factors to assess occupational risks but does not mention the mechanism for performing this procedure.

**Figure 2:**
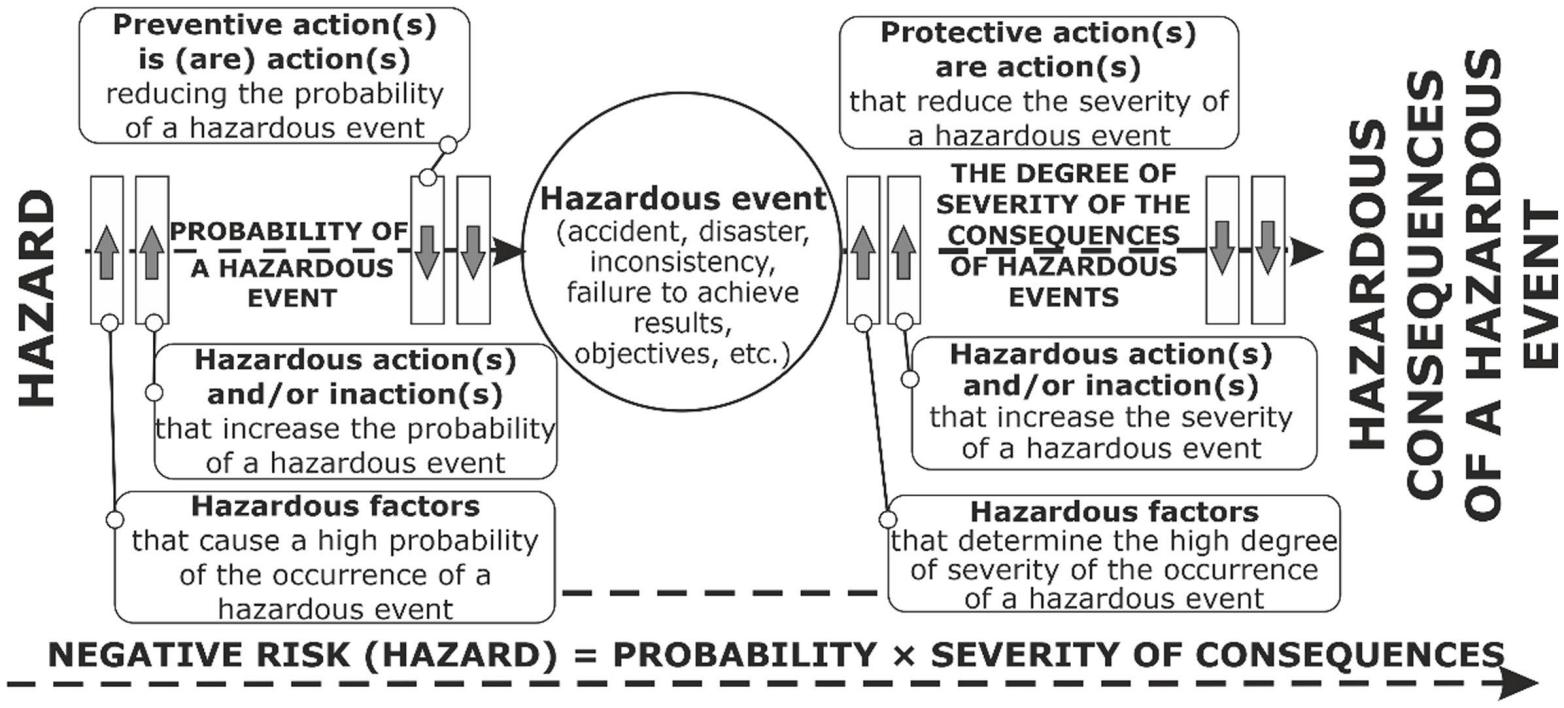
Method of OR hazards management of external and internal environment of the enterprise.

According to the IEC 31010:2019 standard, the “BOW-TIE” method is necessary to establish a cause-and-effect relationship to determine the amount of risk: “hazard–hazardous event consequences” while assessing the probability of the occurrence of a hazardous event and its degree of severity, which is not possible without identifying characteristic hazardous factors of the external and internal environment of the organization, which are related to a specific workplace. Therefore, it is proposed to improve the “BOW-TIE” model, in which, instead of the location of “barriers” [preventive or protective measures and means (Figure 1) that reduce the occurrence of a hazardous event and the severity of the consequences, we provide information about the entire set of negative and safe external and internal factors that increase the probability or severity of the consequences of a hazardous event (Figure 2)].

The basis of the improved “BOW-TIE” method is the calculation of the amount of occupational risk from the combined action of all hazardous factors: physical, chemical, climatic, biological, psychophysiological, ergonomic, technical, organizational, and others, as well as hazardous actions and inactions of employees (errors, malicious intent, professional incompatibility, etc.) in the organization (Figure 2). It should be recalled that according to the ISO 31010 standard, the limitations of this method include the inability to study the occurrence of a hazardous situation in the absence of a clear relationship with the hazard, especially when there is more than one.

As a result, the effectiveness of the occupational risk management process is improved due to a comprehensive consideration of all hazardous external and internal factors or hazardous actions and inactions.

At the first stage of OR evaluation, there is a need for a clear understanding of OHSMS terms in accordance with the requirements of the ISO 45001:2018 standard. It is necessary to clearly distinguish what is a “hazard” and what are “influence factors”—a hazardous factor that increases the probability of a hazardous event.

**Figure 3:**
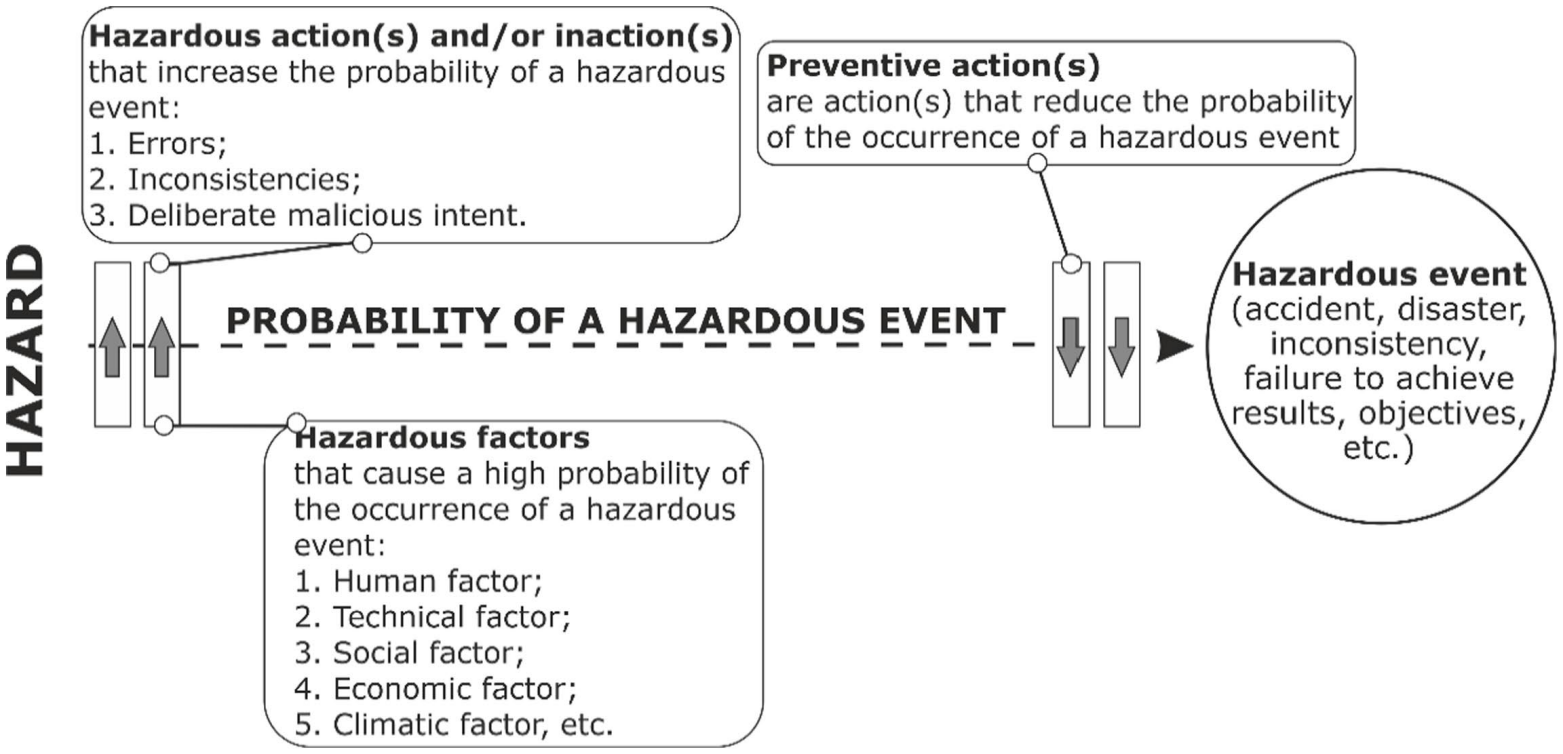
Method of OR hazards management of external and internal environment of the enterprise from the point of the probability of the occurrence of a hazardous event.

**Figure 4:**
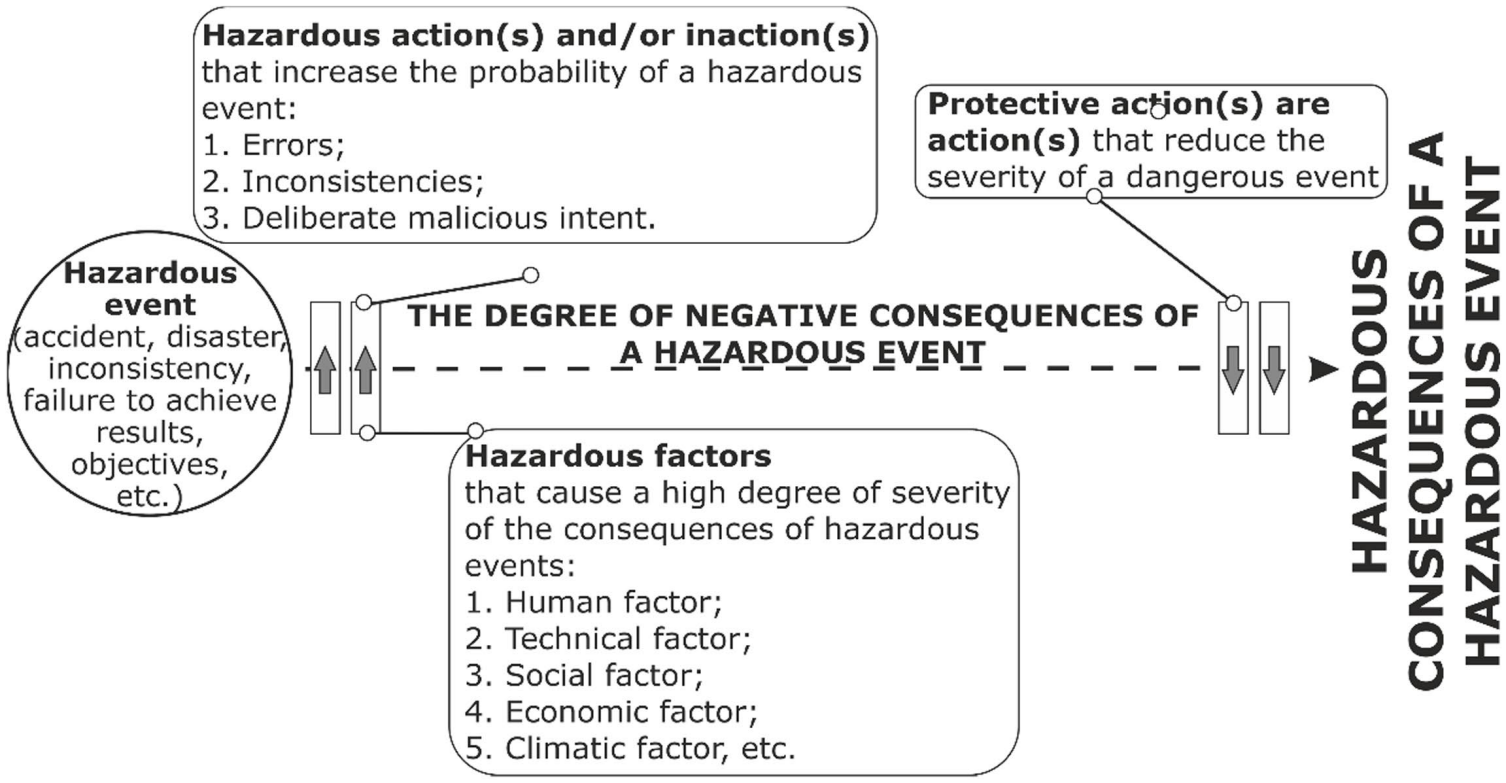
Method of OR hazards management of external and internal environment of the enterprise from the point of reduction of the degree of severity from the occurrence of a hazardous event.

According to ISO 45001:2018, a hazard is defined as any source with the possibility of causing injury and deterioration of health. A hazardous factor increases the probability of occurrence of a hazardous event (incident) or/and also influences and increases the severity of injury and deterioration of health that may be caused by a hazardous event (incident). That is, in order to calculate the probability of occurrence of a hazardous event and its severity, it is necessary to identify all hazardous factors (Figures 3, 4):

- Increase the probability of the occurrence of a hazardous event.
- Increase the degree of severity of a hazardous event.
- Increase both the probability of the occurrence of a hazardous event and the severity of its consequences.

In addition, it is proposed to increase the effectiveness of OR management by taking into account hazardous actions and without the action of the employee (39), which are as follows:

- Increase the probability of the occurrence of a hazardous event.
- Increase the degree of severity of a hazardous event.
- Increase the probability of occurrence of a hazardous event, as well as the degree of severity.

Hence, there is a need to improve the OR management process itself. In addition, it is proposed to identify low environmental factors of the enterprise, and hazardous actions and without the actions of employees. That is, in order to calculate the OR of hazard—*j*, we first identify all external and internal HF, hazardous actions. Next, we conduct a risk assessment for each hazardous factor (40, 41):

*Rп_j_n* = *Вп_j_n* × *Тп_j_n*.

Where *Rп_j_n* is occupational risk of a hazardous event from hazard *j* taking into account hazard factors *n*; *Вп_j_n* is the probability of the occurrence of a hazardous event from hazard *j* under the influence of a hazardous factor *n*; Tпjn is degree of severity of consequences from a hazardous event from hazard *j* under the influence of a hazardous factor *n*.

**Figure 5:**
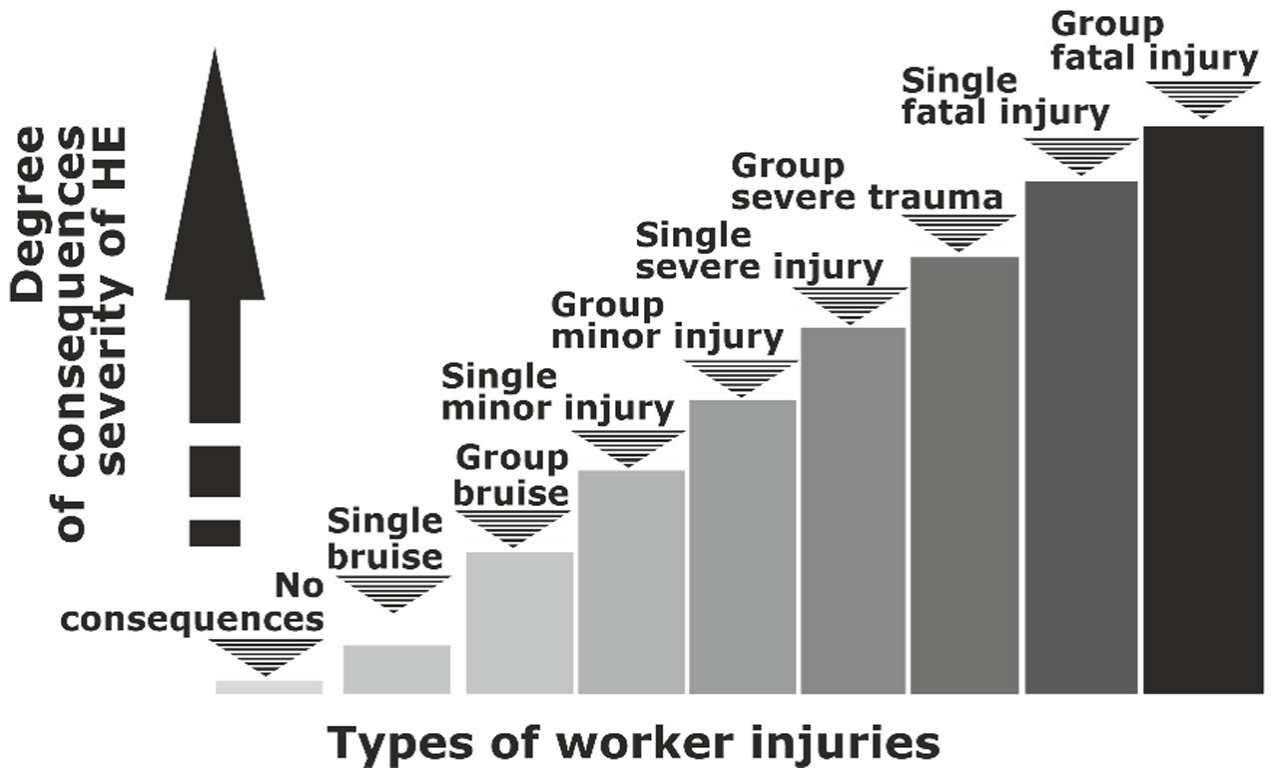
Degrees of severity of consequences of HE according to types of injuries of workers.

To determine the probability of the occurrence of a hazardous event from hazard j, it is important to use statistical data that will become the basis for substantiating the point scale. Determination of the degree of severity of the consequences is based on the classification of types of injuries (Figure 5).

**Figure 6:**
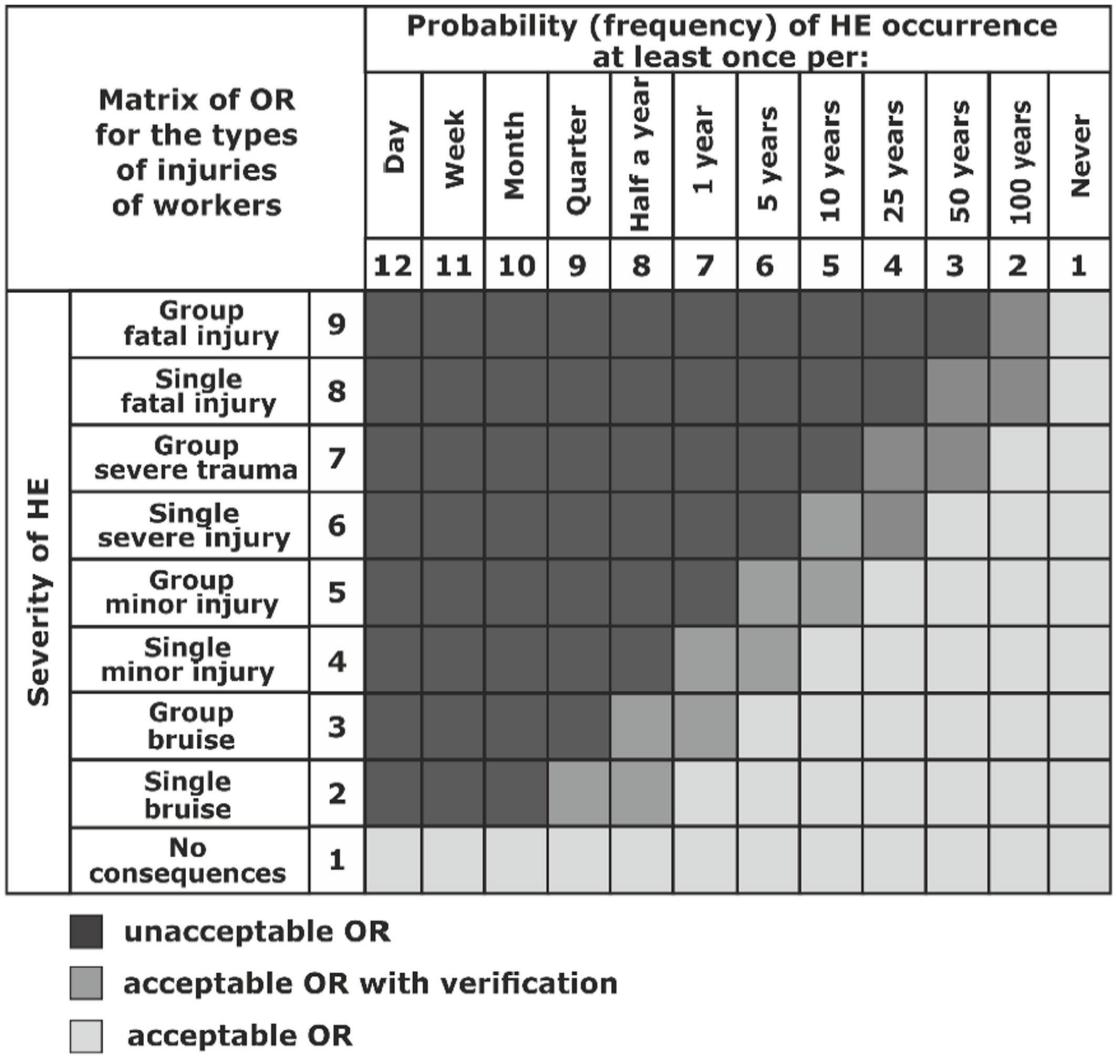
Form of the matrix for determining the value of OR by hazardous factors considering of the type of injuries of workers.

To estimate the value of the OR, a special matrix with a scale of 9 × 12 (Figure 6) is proposed as an example, which is determined on the one hand by the number of negative consequences and, on the other hand, by the probability of the occurrence of the HE.

**Figure 7:**
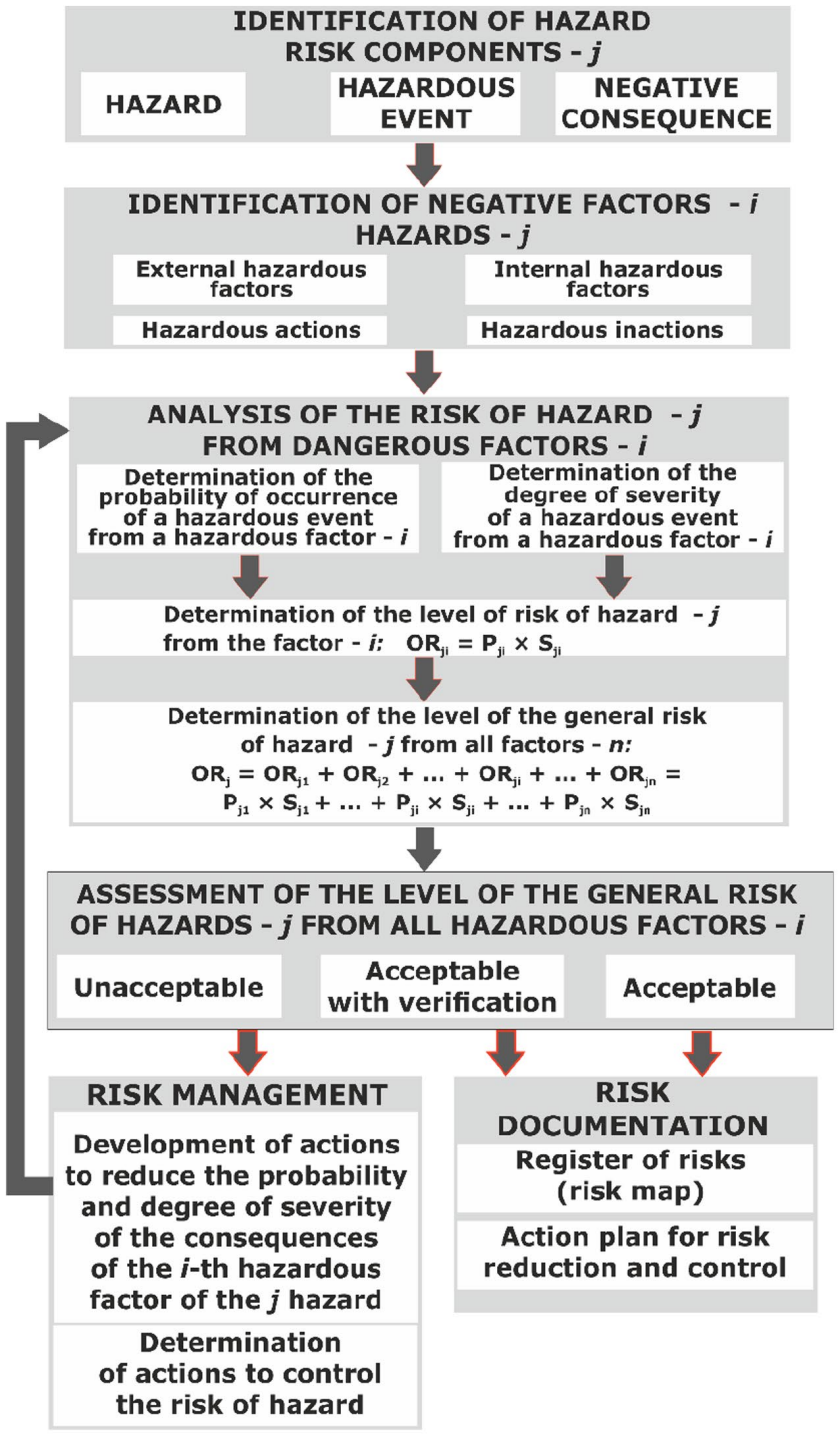
Process of OR managing with additional identification of the HF of enterprise environment and hazardous actions and inaction with the determination of the general OR of the hazard level.

In order to determine the points for the calculation of the occupational risk for each hazardous factor, several experts are invited, the results of whose work are averaged and entered in Table 2, which represents the form of the OR hazard map *j* according to the proposed OR management process (Figure 7).

**Table 2:** Identification of hazards, HF, analysis and assessment of hazard ORs *j* provided that hazard factor of ORs are acceptable and total OR is not acceptable.

Identification			Identification of HF, hazardous actions and inactions	The primary analysis is determination of the OR level for each HF and the overall OR hazard				Preventive and protective actions to reduce the primary OR *j* – hazard from *i* – HF	Final analysis taking into account actions to reduce OR *j* hazard			The final assessment of OR *j* hazard from *i* – HF
Hazard	Hazardous event	Negative consequence	Impact on the probability of occurrence of a hazardous event and/or on the severity of the consequences of a hazardous event from HF	Probability of occurrence of a hazardous event from a hazardous factor - *i*	Severity of occurrence of hazardous event from HF - *i*	Level of OR from HF - *i*	The primary assessment of the negative OR of hazard *- j* for each HF *- i* and the total OR of hazard *- j*		Probability of occurrence of a hazardous event	The degree of severity of the consequences of a hazardous event	Negative OR	
Hazard at the workplace	An accident during the performance of production activities	Injuries, occupational diseases that are the result of an accident	HF_1_	Pe_j1_	Se_j1_	OR_HFj1_	Acceptable or Unacceptable	Preventive and/or protective action j_1_	P_HEj1_	Sc_j1_	ORк_j1_	Acceptable
			HF_2_	Pe_j2_	Se_j2_	OR_HFj2_		−−//−− j_2_	P_HEj2_	Sc_j2_	ORк_j2_	
			HF_3_	Pe_j3_	Se_j3_	OR_HFj3_		−−//−− j_3_	P_HEj3_	Sc_j3_	ORк_j3_	
			HF_4_	Pe_j4_	Se_j4_	OR_HFj4_		−−//−− j_4_	P_HEj4_	Sc_j4_	ORк_j4_	
			HF_5_	Pe_j5_	Se_j5_	OR_HFj5_		−−//−− j_5_	P_HEj5_	Sc_j5_	ORк_j5_	
			HF_6_	Pe_j6_	Se_j6_	OR_HFj6_		−−//−− j_6_	P_HEj6_	Sc_j6_	ORк_j6_	
			HF_7_	Pe_j7_	Se_j7_	OR_HFj7_		−−//−− j_7_	P_HEj7_	Sc_j7_	ORк_j7_	
			HF_8_	Pe_j8_	Se_j8_	OR_HFj8_		−−//−− j_8_	P_HEj8_	Sc_j8_	ORк_j8_	
			HF_9_	Pe_j9_	Se_j9_	OR_HFj9_		−−//−− j_9_	P_HEj9_	Sc_j9_	ORк_j9_	
			HF_10_	Pe_j10_	Se_j10_	OR_HFj10_		−−//−− j_10_	P_HEj10_	Sc_j10_	ORк_j10_	
			HF_11_	Pe_j11_	Se_j11_	OR_HFj11_		−−//−− j_11_	P_HEj11_	Sc_j11_	ORк_j11_	
			….	…	…	…		…	…	…	…	
			HF*_i_*	Pe_ji_	Se_ji_	OR_HFji_		−−//−− j_i_	P_HEji_	Sc_ji_	ORк_ji_	
			…	…	…	…		…	…	…	…	
			HF*_n_*	Pe_jn_	Se_jn_	OR_HFjn_		−−//−− *j_n_*	P_HEjn_	Sc_jn_	ORк_jn_	
			Overall primary negative OR of hazard *j* from all *n* HF	OR_HFj_ = ∑Pe_jn_ × Se_jn_				Overall final negative OR of hazard *j* from all *n* HF	ORк_j_ = ∑P_HEjn_ × Sc_jn_			

The next step is to determine the value of the occupational risk of hazard from the combined effect of all risks from hazardous factors associated with this hazard according to the formula:

*Rп_j_* = *Rп*_*j*1_ + … + *Rп_j_n.*

Where *R*пj is the total occupational risk from hazard *j*; *Rп*_*j*1_ is occupational risk from the first hazardous factor; and *Rп_j_n* is occupational risk from the *n*-th hazardous factor.

Next, we assess the total risk and assess its level of acceptability or unacceptability according to the criteria listed in Table 3.

**Table 3:** Acceptance criteria of (negative) OR of hazard.

Assessment of OR	Score
Acceptable slight negative OR	From 0 to 25
Acceptable (with verification) negative OR	From 25 to 50
Unacceptable negative OR	From 50 to 100

## 3 Results

Based on the requirements of the ISO 45001:2018 standard, external and internal HFs (hazards, threats or opportunities) of the working environment at the enterprise can be determined using one of the acceptable methods given in Table 1. In addition to examining hazards in the employees’ workplaces themselves, attention should be paid to the organization’s policy, planning processes in management systems, production technology, and other processes that form the organization’s “environment.”

**Figure 8:**
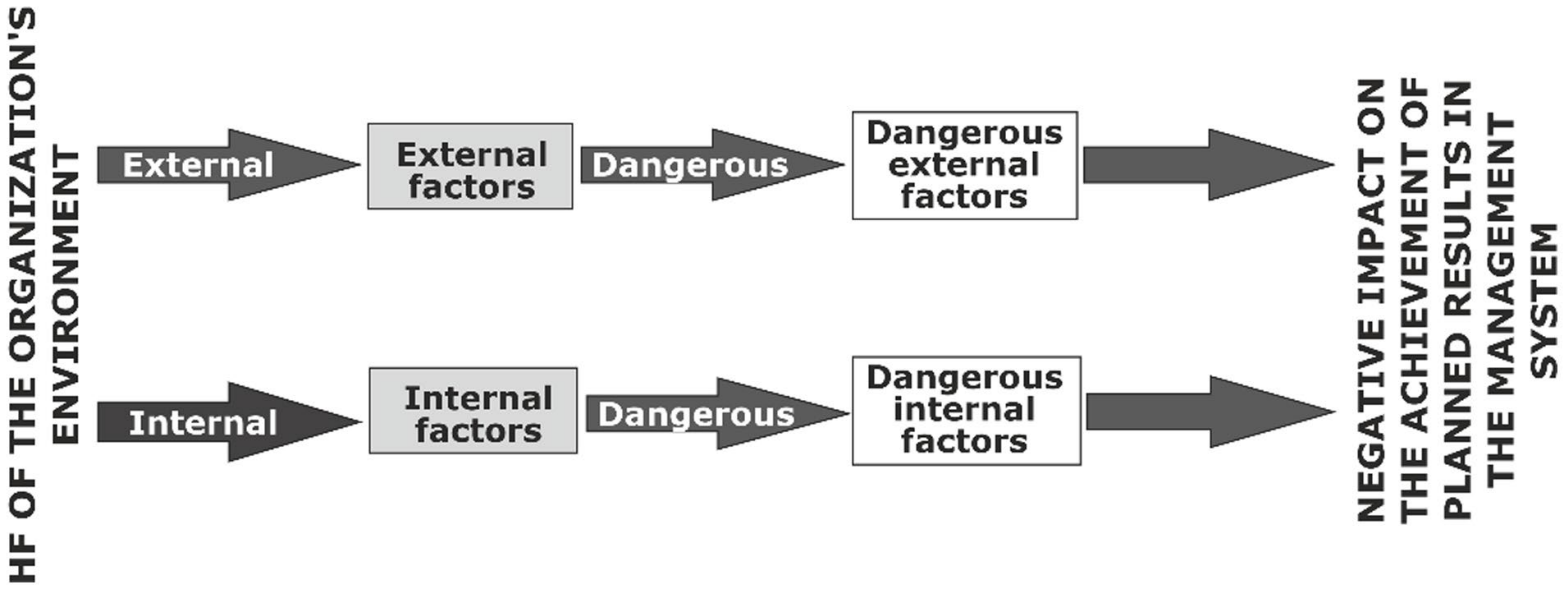
Interrelationship of various factors of the enterprise environment and their influence on the achievement of planned results.

Sources of information about external HF of enterprises can be information posted on the Internet: research, publications in periodicals, news sites, and official sites of state bodies. Information about the state of the internal environment of the enterprise can be obtained from reports on the effectiveness of the OHSMS at the enterprise, internal audits, results of self-assessment, minutes of meetings of managers of various levels, etc. It is important to understand that these HFs have an interrelated effect on the achievement of planned results, which leads to the need to identify OR factors that strengthen/weaken their effect (Figure 8).

Based on the analysis performed, we form a register of hazardous factors by relevant groups (42): organizational, technical, operational, ergonomic, environmental, hygienic, psychosocial, military, and others. The formed register makes it possible to determine the most significant hazardous factors for each emergency situation. This can be done by different methods such as Monte Carlo simulation (43) and expert methods such as fuzzy set theory and fuzzy AHP (44, 45).

Considering different variants of the value of OR, which arise from external and internal HF, hazardous actions and inactions, together allow us to determine the value of the total OR of hazard *j*.

**Figure 9:**
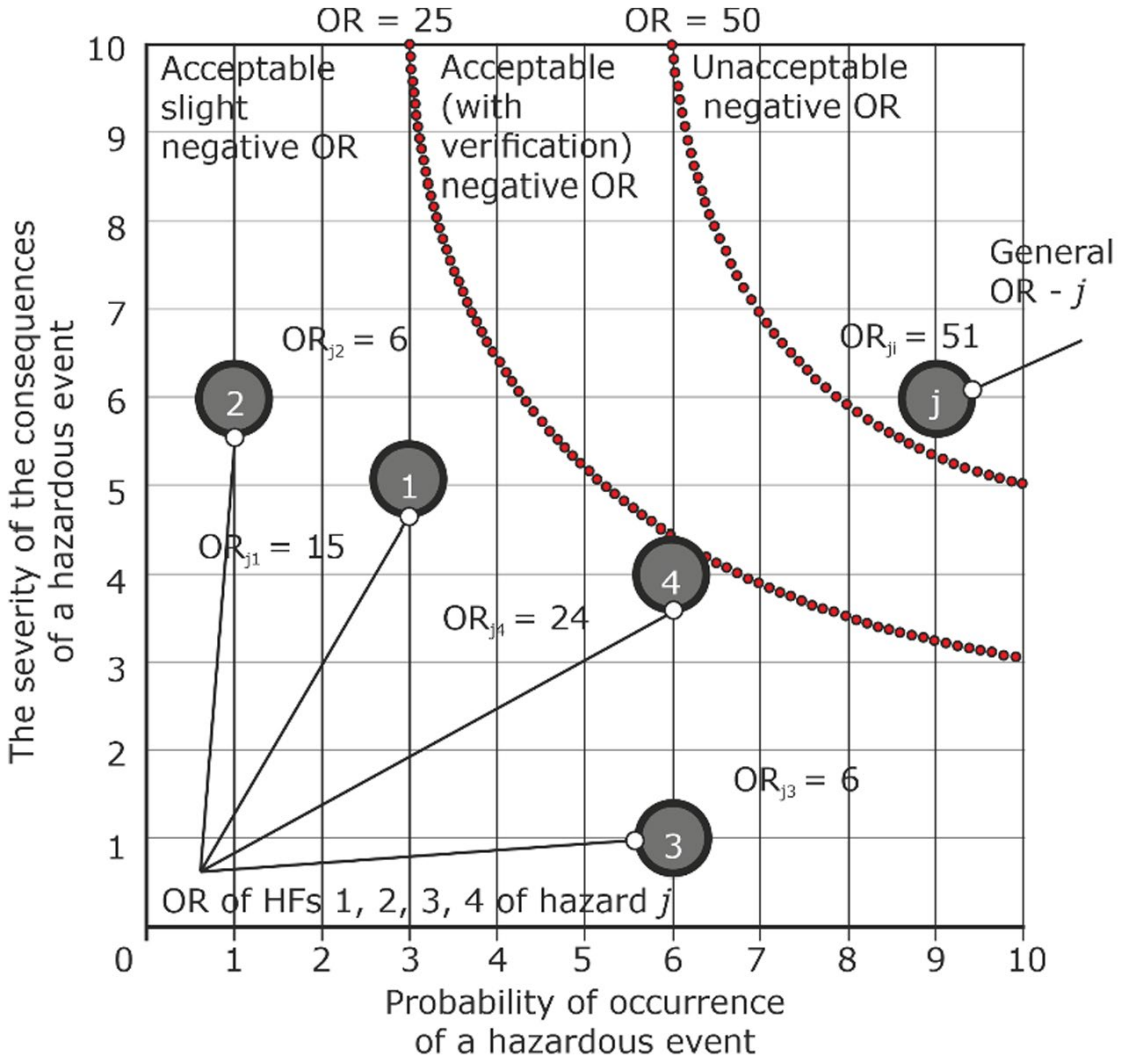
Graphical presentation of assessment of OR hazard j under the condition that the OR of hazardous factors are acceptable, and the general OR is not acceptable.

Therefore, when calculating the value of the total OR, situations may arise when all the ORs from HF are acceptable, and the total OR from the hazard is not acceptable (Table 4; Figure 9). In this case, there is a need to stop the production operation to eliminate or reduce the impact from several detected HFs. Such a situation requires a careful analysis of the impact of the most critical HF.

**Table 4:** Hazard identification, HF, OR of hazard *j* analysis and assessment.

Identification			Identification of HF, hazardous actions and inactions	Primary analysis is determination of the OR level for each HF			
Hazard	Hazardous event	Negative consequences	Impact on the probability of the occurrence of a hazardous event and/or on the severity of the consequences of a hazardous event from HF	Probability of the occurrence of a hazardous event from HF - *i*	The degree of severity from the occurrence of a hazardous event from HF - *i*	OR level from HF - *i*	Primary assessment of hazard OR - *j* by HF - *i* and overall hazard OR - j
*Analysis condition: ORs from HF are acceptable, and overall OR is unacceptable*							
Hazard	Accident	Injury	HF_1_	P_Ej1_ = 3	S_Ej1_ = 5	ORп_j1_ = 15	Acceptable
			HF_2_	P_Ej2_ = 6	S_Ej2_ = 1	ORп_j2_ = 6	Acceptable
			HF_3_	P_Ej3_ = 1	S_Ej3_ = 6	ORп_j3_ = 6	Acceptable
			HF_4_	P_Ej4_ = 6	S_Ej4_ = 4	ORп_j4_ = 24	Acceptable
			…	…	…	…	…
			General primary OR	ORп_j_ = 15 + 6 + 6+ 24 = 51			Unacceptable
*Analysis condition: ORs from HF are acceptable, and overall OR is acceptable with verification*							
Hazard	Accident	Injury	HF_1_	P_Ej1_ = 3	S_Ej1_ = 5	ORп_j1_ = 15	Acceptable
			HF_2_	P_Ej2_ = 1	S_Ej2_ = 2	ORп_j2_ = 2	Acceptable
			HF_3_	P_Ej3_ = 2	S_Ej3_ = 3	ORп_j3_ = 6	Acceptable
			HF_4_	P_Ej4_ = 6	S_Ej4_ = 4	ORп_j4_ = 24	Acceptable
			…	…	…	…	…
			General primary OR	ORп_j_ = 15 + 2 + 6+ 24 = 47			Acceptable with verification
*Analysis condition: ORs from HF are acceptable, and overall OR is acceptable too*							
Hazard	Accident	Injury	HF_1_	P_Ej1_ = 3	S_Ej1_ = 2	ORп_j1_ = 6	Acceptable
			HF_2_	P_Ej2_ = 1	S_Ej2_ = 1	ORп_j2_ = 1	Acceptable
			HF_3_	P_Ej3_ = 2	S_Ej3_ = 3	ORп_j3_ = 6	Acceptable
			HF_4_	P_Ej4_ = 5	S_Ej4_ = 2	ORп_j4_ = 10	Acceptable
			…	…	…	…	…
			General primary OR	ORп_j_ = 6 + 1 + 6+ 10 = 23			Acceptable
*Analysis condition: the OR from the action of one of the HF is unacceptable, and the overall OR is also unacceptable*							
Hazard	Accident	Injury	HF_1_	P_Ej1_ = 3	S_Ej1_ = 2	ORп_j1_ = 6	Acceptable
			HF_2_	P_Ej2_ = 1	S_Ej2_ = 1	ORп_j2_ = 1	Acceptable
			HF_3_	P_Ej3_ = 2	S_Ej3_ = 3	ORп_j3_ = 6	Acceptable
			HF_4_	P_Ej4_ = 10	S_Ej4_ = 6	ORп_j4_ = 60	Unacceptable
			General primary OR	ORп_j_ = 6 + 1 + 6+ 60 = 73			Unacceptable

**Figure 10:**
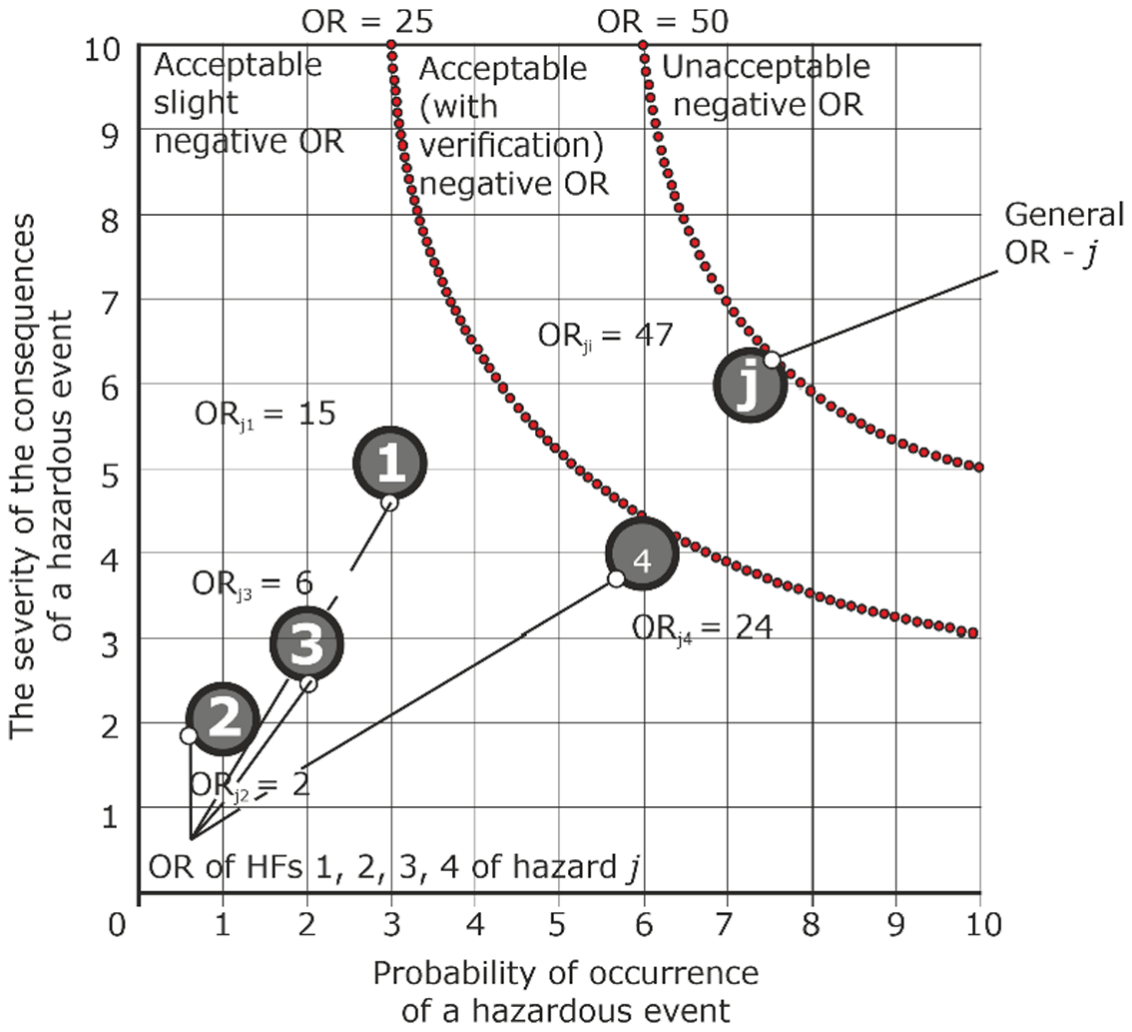
Graphical presentation of assessment of OR hazard j under the condition that the OR of HF is acceptable, and the general OR is acceptable with verification.

The next option may be a situation where the OR from HF action is acceptable, and the total OR is acceptable with verification, i.e., there is a need to control the hazard, HF, and the value of OR, as well as monitor HF or actions of workers that would allow to avoid an unacceptable level OR (Table 4; Figure 10).

**Figure 11:**
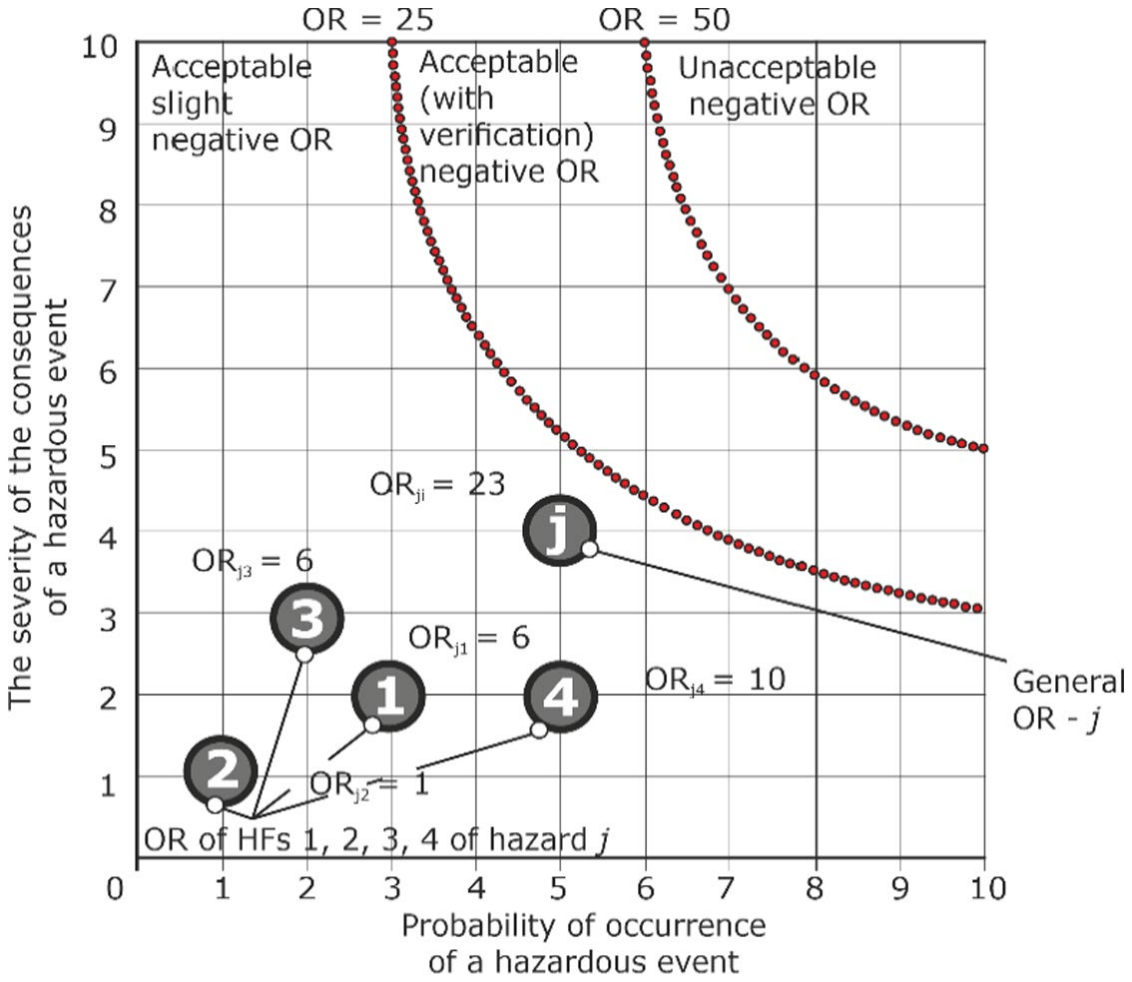
Graphical presentation of assessment of OR hazard j under the condition that the OR of HF is acceptable, and the general OR is acceptable too.

The most acceptable situation is when all ORs from HF and actions are acceptable, which in sum allows us to obtain an acceptable overall OR (Table 4; Figure 11).

**Figure 12:**
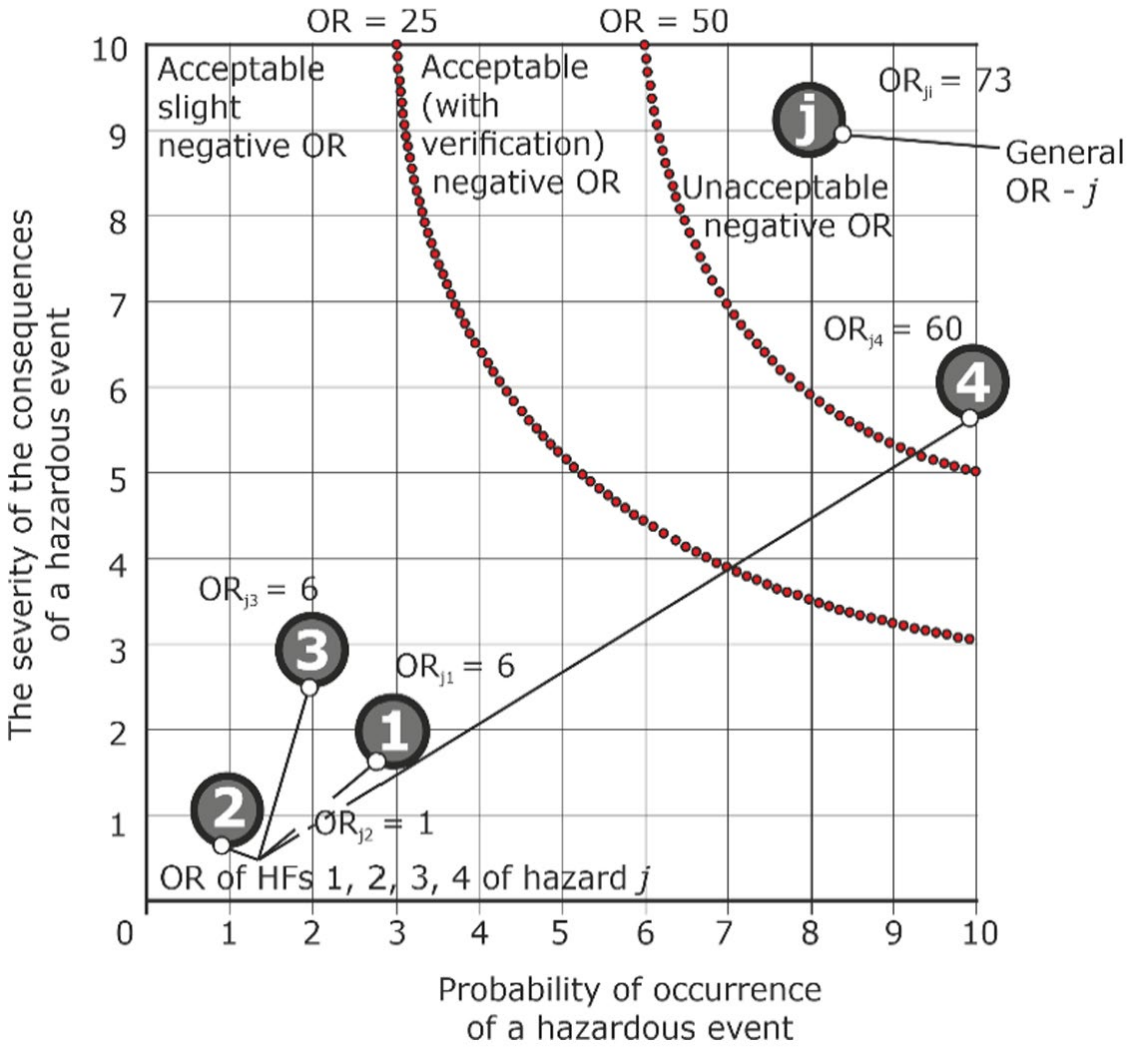
Graphical presentation of assessment of OR hazard j under the condition that the OR of one of HFs is unacceptable, and the general OR is unacceptable too.

The most difficult situation is when there is a condition that one of the ORs from HF action is unacceptable, and the overall OR will also be unacceptable (Table 4; Figure 12). In this case, there is a need to eliminate the detected HF or introduce actions to reduce its influence and conduct a check on the overall value of the OR level.

There is a simple example of an occupational risk assessment of a logger who uses a chainsaw to fell trees in hard-to-reach places (Table 5). Work that is physically demanding (46) is characterized by an uncomfortable body position (47). The main hazard that causes injury is a tree (48). A hazardous event is a falling tree, and the consequences can be injuries of varying severity to the logger’s musculoskeletal system (49). Loggers working outdoors are exposed to a number of different HFs, such as strong wind, inappropriate technical equipment (e.g., personal protective equipment), hygienic hazards [vibration (49), noise (50)], ergonomic hazards—unnatural or uncomfortable working position (48, 51), human factors—psychological inconsistency or health problems.

**Table 5:** Assessing the OR using the example of a logger taking into account the HF impact.

Identification			Identification of HF, hazardous actions and inactions	Primary analysis is determination of the OR level for each HF			
Hazard	Hazardous event	Negative consequences	Impact on the probability of the occurrence of a hazardous event and/or on the severity of the consequences of a hazardous event from HF	Probability of the occurrence of a hazardous event from HF - *i*	The degree of severity from the occurrence of a hazardous event from HF - *i*	OR level from HF – *i*	Primary assessment of hazard OR - *j* by HF - *i* and overall hazard OR - j
Tree	Tree falling	Injury of the musculoskeletal system	Inappropriate physical health of the logger	2	5	10	Acceptable
			Inadequate mental health status of the logger	4	5	20	Acceptable
			Inappropriate environmental conditions – strong wind	2	4	8	Acceptable
			Inadequate technical equipment - inappropriate personal protective equipment	1	4	4	Acceptable
			Inappropriate hygienic conditions: increased noise, vibration	4	3	12	Acceptable
			General primary OR	ORп_j_ = 10 + 20 + 8+ 4 + 12 = 54			Unacceptable

In the above example, each component of the total (cumulative) risk does not exceed acceptable limits, but if certain HFs occur together at any point in time, this will result in unacceptable risk, requiring a review of the impact of each HF. If a traditional method, such as a BOW-TIE, is used to analyze the occupational risk of a logger, each individual hazard will not exceed the critical risk level. This risk assessment often leads to the error that additional precautions are not necessary. It is the total HF value of an unacceptable level that requires again reconsidering the influence of each HF and reducing their influence. In the above example, there is a need to find precautionary measures to reduce the entry of loggers with an inappropriate psychological state into the workplace. At least, we have an understanding of the need for additional introduction of precautionary and protective measures.

## 4 Discussion

A more advanced approach to the OR management process is proposed, which takes into account the influence of external and internal HFs on the probability of a hazardous event occurring and the severity of consequences by summing up the risks from all hazardous external and internal factors in the workplace, given the hazardous actions and inactions of employees.

Its difference from the BOW-TIE method is the ability to take into account the influence of a combination of HFs when analyzing cause-and-effect relationships, which can increase the probability of a hazardous event occurring. At the same time, obstacles that are determined through the hierarchy of precautionary measures (52–54) are reflected in the proposed model as “precautionary actions” that include the strengths of the production environment.

The proposed improved approach to the OR management process allows for a more thorough analysis of external and internal HF, which affect the probability of the occurrence of a hazardous event or the severity of its consequences and therefore ensures the improvement of business process management systems at the enterprise. We should note that any enterprise to ensure effective operation needs to take into account the wishes of interested parties, identify weak points in technology, management systems (55). For this, it is important to ensure a response to all hazardous external and internal factors that threaten the production process in order to reduce losses.

This is implemented in the algorithm that considers four different situations for assessing the OR level from exposure to HFs, which provide conditions for the acceptability/unacceptability of the OR level from exposure to both individual HF and their combined effect. This will make it possible to identify significant HFs or their combination, which increases the probability of a hazardous event occurring. In addition, such an approach will reduce data uncertainty due to poor understanding of the phenomena characteristic of the production process, which is achieved by increasing information about the nature of the processes occurring in the systems (56, 57).

The Bayesian network has a similar probabilistic approach to assessing occupational risk (58). The method consists of two parts: a qualitative diagram and a probability distribution including a quantitative part of the analysis (21). In addition, it makes it possible to construct the same cause-and-effect relationships between various HFs that influence the occurrence of a hazardous situation, for example, according to Dempster’s rule of combination (59). However, the use of this method for assessing occupational risks is difficult, since the algorithm requires a large amount of data, which is mostly determined heuristically or restrictions are introduced to ensure the construction of appropriate relationships between graphs (60). Based on the recommendations of the ISO 31010 standard, this method should only be used for risk assessment. While for other steps that are characteristic of risk management, identification of hazards, determination of the probability of a hazardous event occurring, there is a need to use other approaches, for example, the FMEA or HAZOP methods.

The BOW-TIE method was developed specifically for security management (61). The ISO 31010 standard recommends its use at all stages of risk management: identifying threats, analyzing consequences, risk probabilities, and assessing operational risks. In this sense, BOW-TIE analysis is a combination of FTA and ETA (62), and is very popular since it includes both the causes and consequences of an incident scenario (54).

Note that one of the important requirements of the ISO 45001 standard is the involvement of all employees in the risk management process (63), requiring an appropriate universal approach characterized by easy comprehension and a minimum number of additional steps to assess risks. The BOW-TIE method satisfies these requirements well, so it was decided to improve it in order to increase the efficiency of decision-making. In addition, the ISO 31000 standard indicates the need to move from simple to complex (64). Hence, each of the above risk assessment methods exists and can be applied based on the tasks set before the organization, taking into account available resources to ensure the implementation of the precautionary or protective measures taken to preserve the life and health of the employee and to avoid emergency situations and accidents.

To manage the OR, it is additionally recommended to conduct an analysis of the external and internal environments of the enterprise in order to determine the HFs that affect the probability of the occurrence of a hazardous event and or the degree of severity of the consequences of a hazardous event. To determine the value of the OR of the hazard, it is taken as the sum of the OR from all external and internal HFs, affecting the probability of occurrence of a hazardous event. This approach is based on the assumption that OR is primarily financial losses; in this case, there is an opportunity to determine the total value as the sum of losses from the occurrence of a hazardous event (65, 66).

OR management methods consider the cause-and-effect chain “hazard-hazardous event-loss.” However, the proposed OR management method shows that the occurrence of a hazardous event is possible not only in the presence of hazard but also in conditions for the occurrence of a hazardous event. They are the HFs set, which are associated with negative human, technical, organizational, and other HFs belonging to HFs of the internal environment of the enterprise, as well as factors of the external environment of the enterprise such as season, presence of precipitation, and time of day. At the same time, it is proposed to consider the cause-and-effect chain “hazard-hazardous event-loss” with the influence of HF on the probability of the occurrence of a hazardous event and/or the degree of severity. It is proposed to define OR of hazard as the sum of OR of all HF of a hazardous event.

The shortcomings of this method, which require further research, include the inability to take into account the variability of HFs over time (67, 68). The analysis of various emergency situations and accidents shows (69–71) that the main reason for the occurrence of a hazardous situation is the sequence or simultaneous occurrence of a certain number of hazardous factors in a certain period of time. At the same time, HFs can have different impact on the probability of a hazardous event occurring when an employee performs production tasks, which is conditioned by the presence of strong points in the production environment (72). In particular, this is the competence of the employee, the periodic occurrence of noise or vibration in the workplace, the appearance of a minor technical malfunction in hard-to-diagnose equipment, etc. All this requires the use of combinatorics to identify the most probable combinations of hazardous factors that have a significant impact on the hazardous situation manifestation.

## 5 Conclusion

An improved management process of OR hazard has been developed, taking into account the HF of the internal and external environment of the enterprise, which increases the probability of the occurrence of a hazardous event and the severity of the consequences, provided that the level of OR is determined as acceptable, unacceptable, or acceptable with verification. It is suggested that during OR management, an additional analysis of the external and internal environments of the organization is carried out to determine HF, which affects the probability of the occurrence of a hazardous event and the degree of severity of the consequences of a hazardous event. The process of OR management determines the value of OR of hazard as the sum of OR from all external and internal HF, which affects the increase in the probability of the occurrence of a hazardous event and its severity of consequences. Four different situations were considered, regarding the calculation of the total value of OR, and an algorithm and the form of a map for hazard identification and HF estimation of the OR of the hazard were proposed.

The proposed approach to OR management when analyzing the cause-and-effect relationship between a hazard and a hazardous event makes it possible to identify significant HFs, the reduction of which will allow a better substantiation of the appropriateness of precautions in the employee’s workplace.

## Data availability statement

The raw data supporting the conclusions of this article will be made available by the authors, without undue reservation.

## Author contributions

OB: Funding acquisition, Project administration, Writing – original draft. VT: Conceptualization, Investigation, Supervision, Writing – original draft. MO: Conceptualization, Investigation, Validation, Writing – original draft. AP: Conceptualization, Methodology, Writing – original draft. SC: Formal analysis, Investigation, Methodology, Supervision, Writing – original draft, Writing – review & editing. OY: Formal analysis, Methodology, Validation, Writing – original draft. OD: Formal analysis, Investigation, Methodology, Visualization, Writing – original draft. VL: Funding acquisition, Writing – original draft, Writing – review & editing.
